# Accessibility and Applicability of Currently Available e-Mental Health Programs for Depression for People With Poststroke Aphasia: Scoping Review

**DOI:** 10.2196/jmir.9864

**Published:** 2018-12-04

**Authors:** Stephanie Jane Clunne, Brooke Jade Ryan, Annie Jane Hill, Caitlin Brandenburg, Ian Kneebone

**Affiliations:** 1 School of Health and Rehabilitation Sciences The University of Queensland Brisbane Australia; 2 Discipline of Clinical Psychology Graduate School of Health University of Technology, Sydney Ultimo Australia

**Keywords:** aphasia, stroke, depression, mental health, internet, technology, access to health care

## Abstract

**Background:**

Depression affects approximately 60% of people with aphasia 1 year post stroke and is associated with disability, lower quality of life, and mortality. Web-delivered mental health (e-mental health) programs are effective, convenient, and cost-effective for the general population and thus are increasingly used in the management of depression. However, it is unknown if such services are applicable and communicatively accessible to people with poststroke aphasia.

**Objective:**

The aim of this study was to identify freely available e-mental health programs for depression and determine their applicability and accessibility for people with poststroke aphasia.

**Methods:**

A Web-based search was conducted to identify and review freely available e-mental health programs for depression. These programs were then evaluated in terms of their (1) general features via a general evaluation tool, (2) communicative accessibility for people with aphasia via an aphasia-specific communicative accessibility evaluation tool, and (3) empirical evidence for the general population and stroke survivors with and without aphasia. The program that met the most general evaluation criteria and aphasia-specific communicative accessibility evaluation criteria was then trialed by a small subgroup of people with poststroke aphasia.

**Results:**

A total of 8 programs were identified. Of these, 4 had published evidence in support of their efficacy for use within the general population. However, no empirical evidence was identified that specifically supported any programs’ use for stroke survivors with or without aphasia. One evidence-based program scored at least 80% (16/19 and 16/20, respectively) on both the general and aphasia-specific communicative accessibility evaluation tools and was subject to a preliminary trial by 3 people with poststroke aphasia. During this trial, participants were either unable to independently use the program or gave it low usability scores on a post-trial satisfaction survey. On this basis, further evaluation was considered unwarranted.

**Conclusions:**

Despite fulfilling majority of the general evaluation and aphasia-specific evaluation criteria, the highest rated program was still found to be unsuitable for people with poststroke aphasia. Thus, e-mental health programs require substantial redevelopment if they are likely to be useful to people with poststroke aphasia.

## Introduction

### Background

Aphasia is a language disorder that can impact a person’s ability to understand and produce spoken language, read, write, calculate, and use gestures [1]. It is an acquired neurological condition that results from brain damage and occurs in approximately 30% of first-time strokes [2,3]. The communication changes experienced by individuals with poststroke aphasia may cause social exclusion, diminished social networks, activity limitations, reduced life participation, and lower quality of life [4-6]. Depression affects about one-third of stroke survivors without aphasia [7,8] and approximately 60% of stroke survivors with aphasia 1 year post stroke [3]. Negative emotional outcomes after stroke are associated with disability, lower quality of life, and mortality [8]. Hence, poststroke depression is a pressing clinical issue for those with poststroke aphasia.

Talk-based psychological interventions, such as cognitive behavioral therapy (CBT), are widely recommended in the treatment of depression [9-11]. However, a lack of suitably trained professionals, barriers related to cost, distance to care, transport, physical disability, time constraints, stigma, and impaired mobility make psychological interventions relatively inaccessible to many in need [12-16]. Significant communication disability may also prevent people with poststroke aphasia from participating in traditional talk-based psychological treatments [17]. For example, a recent study found that speech pathologists in the United Kingdom often perceived mental health professionals as being underskilled in working with people with poststroke aphasia and that this was a major barrier to referring aphasia patients onto mental health professionals [17]. It is acknowledged that during face-to-face communication, people with aphasia can make use of gesture, facial expression, sign, tone of voice, etc, to help understand others and to get their own message across. Although these communication supports are not offered by e-mental health interventions, there are still many examples of people with poststroke aphasia successfully accessing Web- and computer-based programs and interventions [18-20].

e-mental health is a digital form of mental health care and may offer a solution to the accessibility issues of face-to-face therapy commonly encountered by the general population. e-mental health services provide treatment and assistance to people suffering from mental illness via digital platforms such as computers and Web-based programs [21]. The term *e-mental health* encompasses both e-mental health *literacy*, which is the provision of information pertaining to the nature and treatment of mental health illnesses, and e-mental health *programs*, which are structured self-help programs designed to treat or prevent mental health disorders via an interactive interface [22].

In the context of e-mental health, accessibility refers to the ease with which patients can utilize the health care service in proportion to their needs, as well as the usability of the actual technology through which that service is provided [23]. The increased accessibility and convenience offered by e-mental health programs may enable patients to surpass the barriers associated with limited services, transportation, time, cost, and stigma [13,21]. This increased accessibility may be especially beneficial for patients with poststroke aphasia because of the high incidence of both mood disorders [8] and physical impairment [24] after stroke.

There is a large body of research that supports the use of e-mental health programs for depression; however, this research is generally limited to patients with depression who are otherwise healthy. Although 1 study reported preliminary evidence for computerized CBT (cCBT) in reducing depressive symptoms in people with traumatic brain injury [25] (a population that may also present with acquired communication deficits including aphasia [26]), patients with “insufficient English language skills” were excluded. Thus, such findings cannot be generalized to people with poststroke aphasia. Furthermore, a recent feasability randomized control trial (RCT) of a cCBT intervention for stroke survivors concluded that guided cCBT could potentially increase the accessibility of psychological support for stroke survivors [27]. However, the participants did not specifically have poststroke aphasia, and the communicative needs of stroke survivors with aphasia differ from those without. To the best of the authors’ knowledge, these are the only 2 studies that have investigated e-mental health programs directly in people with an acquired brain injury. Therefore, it is not yet known whether such services, and the digital technologies through which they are delivered, are communicatively accessible to people with poststroke aphasia.

### Objectives

Previously, a scoping review identified and evaluated currently available e-mental health interventions for depression [28]. This review also acknowledged a lack of e-mental health programs for special populations and recommended that future studies investigate the accessibility needs of such populations so that adequate treatment can be made available to them. To the authors’ knowledge, no study has explored e-mental health treatment in terms of its suitability for people with depression and concurrent poststroke aphasia. This is the aim of this study.

Specific objectives were to (1) evaluate the general features of each program, (2) review the published evidence of each program for the general population and for stroke survivors with and without aphasia, (3) evaluate each program’s communicative accessibility for people with poststroke aphasia, and (4) determine which e-mental health program(s) may be most suitable for people with poststroke aphasia. It should also be noted that this study did not aim to evaluate how psychotherapeutic concepts, such as CBT principles and abstract concepts, were presented to users in the context of a broader psychotherapeutic community.

## Methods

### Scoping Review

In all, 2 previous scoping reviews that evaluated available e-mental health interventions for depression [28] and anxiety [29] within the general population were used as the basis of the methodology for this review. The authors aimed to simulate a Web-based search that would likely be carried out by a person with poststroke aphasia seeking free Web-based treatment for depression.

#### Search Strategy

A Web-based search for e-mental health interventions for depression was conducted in July 2017. Consistent with previous scoping reviews [28,29], the search engine Google was used for the Web-based search. Prior research has found that people often use search engines, particularly Google, when seeking Web-based health information [30,31]. Hence, people with poststroke aphasia are more likely to find publicly accessible e-mental health programs via search engines rather than through academic resources such as journal databases. The Web-based search consisted of 2 stages: (1) a general Web-based search for e-mental health treatment for depression and (2) a Web-based search for e-mental health depression treatments for stroke survivors with and without aphasia. A total of 12 general search terms were used in the first stage of the Web-based search; they consisted of simple, lay keywords and did not include the words “stroke” or “aphasia.” As the most recent E-mental Health Strategy for Australia specifies the Government’s investment in Web-based CBT programs [21], many of these search terms relate to CBT. During stage 2 of the Web-based search, the authors collaborated with an academic advisory group, consisting of clinicians and academics who work with people with poststroke aphasia, to generate a set of search terms they thought a person with poststroke aphasia might use if searching for Web-based treatment for depression. These search terms were then combined with the general search terms in stage 1 that yielded the most results. This resulted in 6 aphasia-specific search terms that were used in stage 2 of the Web-based search. All 18 search terms are included in Textbox 1.

#### Program Screening

Consistent with a previous scoping review [29], the first 25 hyperlinks generated by each search term were screened. This methodology was replicated because it has been found that 75% of users never scroll past the first page of search results [32]. This resulted in 450 hyperlinks being screened. All 450 hyperlinked websites were recorded in an Excel document and, as done in a previous scoping review [29], categorized as (1) websites with e-mental health programs; (2) websites linking to websites with e-mental health programs; and (3) websites with irrelevant content. Irrelevant content included advertisements, scholarly articles, blogs, websites for face-to-face psychology clinics, chatrooms, and support forums. All websites classified as “irrelevant content” and all duplicates identified within the first 2 categories were removed. The remaining hyperlinked websites were then screened using the following inclusion criteria: (1) designed for depressive symptoms in adults; (2) publicly accessible to the general population via the internet; (3) is a structured, self-management program; and (4) free for Australian residents.

**Search terms used in stage 1 and stage 2 of the Web-based search.**

**Stage 1—general search terms**

- Internet therapy for depression
- Internet treatment for depression
- Internet help for depression
- Internet cognitive behavioral therapy for depression
- Web therapy for depression
- Web treatment for depression
- Web help for depression
- Web cognitive behavioral therapy for depression
- Online therapy for depression
- Online treatment for depression
- Online help for depression
- Online cognitive behavioral therapy for depression

**Stage 2—aphasia-specific search terms**

- Online therapy for depression aphasia
- Online therapy for depression after stroke
- Online treatment for depression aphasia
- Online treatment for depression after stroke
- Online cognitive behavioral therapy for depression aphasia
- Online cognitive behavioral therapy for depression after stroke

Websites were excluded if they (1) provided e-mental health literacy only; (2) offered purely Web-based counseling; (3) were designed for specific populations other than stroke survivors with or without aphasia (ie, adults who stutter); (4) were designed exclusively for adolescents and/or young adults (ie, 25 years or younger); or (5) were not available in English. As some programs required users to complete a depression symptom questionnaire to determine whether the program was suitable for the user before creating an account, the authors contacted all program developers asking for research access to the program. Programs were excluded if research access was not granted.

#### Data Extraction and Evaluation of Programs

In all, 3 separate tools were used to collect and evaluate the data from each program: a data extraction form, a general evaluation tool, and then an aphasia-specific evaluation tool. The authors used the data extraction form to extract relevant information and data from each program, which then underwent evaluation using the general evaluation and the aphasia-specific evaluation tools. The categories within both the general and aphasia-specific evaluation tools contained a set of closed choice (“yes” or “no”) questions. A score of 1 was awarded if the question was answered with a “yes”; a score of 0 was awarded if the question was answered with a “no” or could not be evaluated (ie, due to restricted access to the program). Consistent with previous scoping reviews [28,29], the total numerical score for each captured program was converted into a percentage, with higher percentages representing higher levels of criteria fulfillment.

#### Data Extraction

With permission from the authors, the data extraction form used in a previous scoping review [29] was adapted for use in this study (see Multimedia Appendix 1). For each e-mental health program, data were extracted for the following categories: website characteristics (ie, origin, organizational affiliation, general accessibility and credibility), program characteristics (ie, intervention focus, design, and delivery), intervention characteristics (ie, therapeutic approach and intervention features), and empirical evidence for program efficacy within the general population as well as for stroke survivors with and without aphasia.

To determine empirical evidence for each program, the authors scrutinized each website for relevant information and searched the program’s name in each of the following databases: PubMed, Cumulative Index to Nursing and Allied Health Literature (CINAHL), Cochrane Library, and Web of Science. If these methods failed to identify published evidence, the authors contacted the program’s developers to enquire about program efficacy. Data extraction was completed by the first author during July 2017.

#### General Evaluation Tool

The general evaluation tool was used to evaluate each e-mental health program in terms of its general features (ie, website, program and intervention characteristics) and empirical evidence (see Multimedia Appendix 2 for completed form). It was based on the program evaluation criteria used in a previous review [29]. With permission from the authors, the original evaluation tool was adapted by adding in the following questions:

1. Was a mobile app version of the program available?
2. Did the program send completion reminders?
3. Were text-entry fields present?

#### Aphasia-Specific Evaluation Tool

The aphasia-specific evaluation tool was developed by the authors to assess each program in terms of its communicative accessibility for people with poststroke aphasia (see Multimedia Appendix 3 for the completed form). The evaluation criteria within this tool were based on existing aphasia-friendly guidelines for printed materials and accessibility features of related products [33-35], as well as usability considerations for older people (ie, simple menu hierarchies and text that contrasts the color of the background [36]). The aphasia-specific evaluation criteria contain 7 main categories: vocabulary and syntax, screen clarity, formatting, graphics, navigation, interface design, and media type. Vocabulary and syntax were evaluated by determining the readability level of the text, according to the Flesch-Kincaid reading grade levels [37]. This was done electronically by copying and pasting all text from the first module or session of each program into a Microsoft Word document. One program (myCompass; The Black Dog Institute) did not require modules to be completed in a specific order. Therefore, readability was based on the *Tackling Unhelpful Thinking* module, as it was described to be useful for anyone with mild to moderate depression. Text written at level 5 readability or lower was considered to be appropriate for people with poststroke aphasia, as per the Stroke Association’s Accessible Information Guidelines [38]. An example of text written at or below a level 5 readability level when presented as a 3-lined paragraph is as follows: *A stroke can cause aphasia. People with aphasia are still smart. People with aphasia can still solve problems*. Font size was determined by selecting text on the webpage and using the right click *inspect* function. A ruler was used to physically measure the amount of white space between lines of text as presented on a 15.6-inch (40-cm) laptop screen at 100% zoom. Spacing of 4 mm or more was considered adequate for people with aphasia, as it is equivalent to the amount of white space measured between the lines of Times New Roman typeface set at 1.5 line spacing. The presence or absence of aphasia-friendly design characteristics, such as the use of bullet points and numbering to establish key points, the use of headings to make important information stand out, and the use of bolding to highlight important information, was also determined.

### Accessibility Test

#### Participants

Participants were recruited through a speech pathology intervention clinic operating at The University of Queensland. All members attending a weekly aphasia group were invited to participate in the study. People with aphasia who were aged 18 years and older, had sufficient knowledge of English language to participate without a translator, and had adequate vision for reading were invited to participate. The exclusion criteria were as follows: presence of a concomitant progressive neurological condition (eg, dementia) or a concurrent medical condition impacting their mental health (eg, cancer) as confirmed by self-report.

### Materials

#### Computer Use Survey

A computer use survey was used to determine participants’ level of computer use before and after aphasia, as well as their reasons for using a computer. This survey was created for a separate study that explored computer use by people with aphasia [39]. With permission from its publishers, this survey was adapted by asking participants if they currently or previously used a computer for the treatment of mental health difficulties and which programs they used. Furthermore, questions about computerized speech therapy programs and participants’ likes and dislikes about using a computer in general were removed.

#### Observation Form

An observation form was developed to rate each participant’s level of independence using the selected e-mental health program. Independence was rated for 6 main categories: logging in to a premade account, navigating the program, reading and understanding text, completing interactive activities, completing exercises, and finishing the session. The checklist used a 5-point scale where 1=not at all independent, 2=minimally independent, 3=moderately independent, 4=mostly independent, and 5=totally independent.

#### Satisfaction Survey

A satisfaction survey was developed to evaluate participants’ satisfaction with using the e-mental health program, including its accessibility and ease of use. The survey consisted of 16 questions and statements for which participants answered using a 5-point rating scale (1=no, definitely not; 2=no, I don’t think so; 3=neutral; 4=yes, I think so; 5=yes, definitely so). There were also opportunities for participants and their family members to add comments about the program.

#### Procedures

Ethical approval was granted by The University of Queensland Human Research Ethics Committee. All participants provided written informed consent before participation. Participants completed the computer use survey with assistance from their family member, carer, or research assistant, as needed. Individually, each participant then completed 1 module of the selected e-mental health program on a desktop computer. The research assistant observed each participant trialing the e-mental health program while completing the observation form. The research assistant also provided support to the participants by reading aloud informative text, reading aloud instructions, showing participants where to click/type, re-explaining instructions, controlling the mouse, and/or typing for the participants, as required. After trialing the e-mental health program, each participant completed the satisfaction survey with assistance from their family member, carer or research assistant, as needed. Each trial session was video-recorded and rewatched by the research assistant to confirm observations that had been made.

## Results

### Scoping Review

#### Program Selection

**Figure 1:**
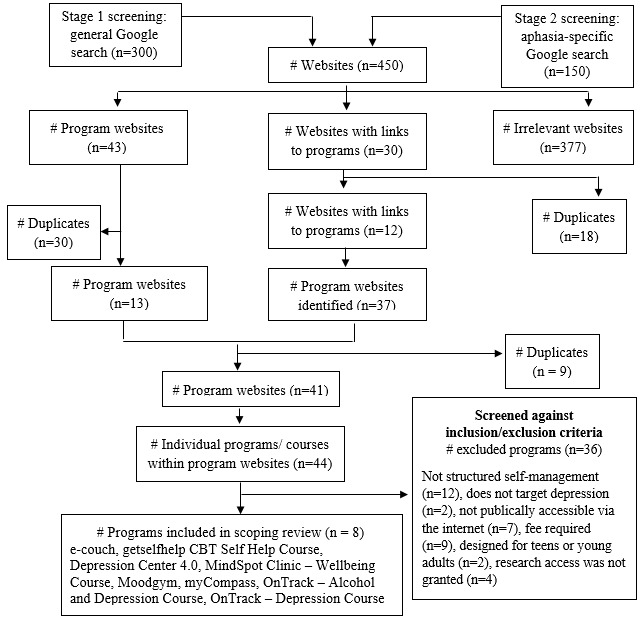
Flow diagram of program selection.

In total, 43 websites with e-mental health programs, 30 websites linking to websites with e-mental health programs, and 377 irrelevant websites were identified. After duplicate and irrelevant websites were removed, 41 websites remained, which yielded 44 individual programs. Of those programs, 8 programs met the inclusion and exclusion criteria. Figure 1 depicts the flow diagram for the program selection process, including reasons for exclusion and the final 8 programs reviewed. The authors were not granted full access to one of the included programs (the Wellbeing Course; MindSpot), and therefore evaluated this program using a “demo” version of the course that is available for practitioners.

#### Data Extraction

An overview of the programs’ website characteristics, program characteristics, and intervention characteristics can be found in Multimedia Appendices 4-6.

Two programs purely targeted depressive symptoms (OnTrack—Depression; Queensland University of Technology, and Depression Center 4.0; Evolution Health). The program OnTrack—Alcohol and Depression (OnTrack—AD; Queensland University of Technology) focused on depression with comorbid alcohol problems. The remaining 5 programs were reported to be designed for people with either depression with or without other conditions, including anxiety, anger, worry, stress and low mood, as well as emotional difficulties related to divorce, separation, bereavement, and loss. One program (myCompass; Black Dog Institute) specified user suitability criteria, while the remaining programs gave general information about whom the program would be appropriate for. All programs specified that they were designed for adult users, with 2 programs defining a specific age range for users; one being between 26 and 64 years (the Wellbeing Course) and the other being between 18 and 75 years (myCompass). In its program suitability criteria, myCompass specified that users should be able to “read English with ease.” Furthermore, 3 programs had the option to send completion reminders to users via email or text message (myCompass, OnTrack—AD, and OnTrack—Depression).

All but 2 programs provided unguided therapy. One of the guided programs provided users with free support from a trained therapist via telephone or email (the Wellbeing Course). The user could choose to receive this support weekly or could choose to contact the therapist whenever he or she wished. It was not clear whether the therapist support was optional or whether the user had to contact the therapist at some point, and an attempt to contact the program’s developers was unsuccessful in obtaining this information. The other guided program (Depression Center 4.0) included a *Questions to the Expert* section, where users could submit questions to be answered by a clinical psychologist. This program also contained forums that were moderated by health educators.

All programs claimed to employ CBT, an approach that helps people to identify and challenge negative thought patterns and learn practical self-help strategies [9]. In addition, 3 programs also used interpersonal therapy (e-couch by e-hub Health; Moodgym by e-hub Health; and myCompass), a talk-based, time-limited treatment approach that targets symptom resolution, increased social support, and improved interpersonal functioning [40]. Furthermore, 2 programs employed problem-solving therapy (e-couch and myCompass), a treatment approach that focuses on constructive problem-solving skills and attitudes to help individuals cope more effectively with life’s stressors [41]. Positive psychology, which focuses on one’s positive experiences and qualities rather than on situations and qualities that cause suffering, was also used by 1 program (myCompass) [42], whereas 2 programs (OnTrack—AD and OnTrack— Depression) included meditational mindfulness, an approach that uses aspects of meditation to facilitate self-regulation of attention and help users adopt an accepting outlook on their experiences [43].

### Empirical Evidence

#### Empirical Evidence for Program Efficacy Within the General Population

An initial search through all the programs’ websites for research evidence identified 2 published articles reporting on program efficacy for depression for Moodgym [44] and myCompass [45]. A database search in PubMed, CINAHL, Cochrane Library, and Web of Science yielded 9 more published research articles investigating e-couch [46-48] and the Wellbeing Course [49-54]. The authors contacted the developers of the remaining programs to enquire about program efficacy. In response, the authors received unpublished data investigating the efficacy of 2 programs (OnTrack—AD and OnTrack—Depression; personal communication by J. Connolly, August 2017). Only the highest level of evidence for each program was reviewed according to the National Health and Medical Research Council’s evidence hierarchy [55]. For example, if an RCT was identified for a program, lower level evidence (eg, a case-control study) for that same program was not reviewed. There was, however, an exception to this, whereby an RCT evaluating Moodgym was reviewed despite there being a meta-analysis available for this program, as the RCT included participants with stroke [56]. Table 1 outlines the highest level of published evidence for each program.

#### Empirical Evidence for Program Efficacy After Stroke and/or for People With Poststroke Aphasia

No studies were found that evaluated efficacy of use specifically for stroke survivors with or without aphasia. Due to a technical error in the research portal, 1 RCT included in Moodgym’s meta-analysis unintendedly recruited a number of participants who were later found to have a brain injury or stroke or who were already receiving CBT treatment for bipolar disorder [56]. This RCT used an attention-control (e-mental health literacy package) and reported improvements in depression in both the intervention and attention-controlled groups but no significant differences between groups [56]. It is unknown how many stroke survivors participated, as they could not be differentiated from those with brain injury or those receiving CBT for bipolar disorder (personal communication by J. Schneider, September 2017). One of the RCTs for e-couch included participants with a previous doctor’s diagnosis of heart disease, stroke, or hypertension [46]. This RCT reported improvements in depressive symptoms after completing the e-couch program [46]. A total of 15 out of 562 participants were stroke survivors, but there were no data regarding the inclusion of people with aphasia (personal communication by N Glozier, September 2017). These were the only studies that included participants with known long-term health conditions. Neither of these studies completed subgroup analysis.

### Results From the General Program and Aphasia-Specific Communicative Accessibility Evaluation Tools

The overall scores and corresponding percentages from the general evaluation and aphasia-specific evaluations are outlined in Table 2. General program evaluation scores for each criterion within the general evaluation tool and aphasia-specific communicative accessibility evaluation tool are available in Multimedia Appendices 2 and 3, respectively. General program evaluation scores ranged from 26% (5/19; getselfhelp; Carol Vivyan) to 84% (16/19; OnTrack—AD, Moodgym, and myCompass; mean: 71% [SD 18.1]). Aphasia-specific communicative accessibility evaluation scores ranged from 55% (11/20; getselfhelp) to 85% (17/20; OnTrack—AD; mean: 72% [SD 9.3]). As Moodgym was the only evidence-based program to score at least 80% on both the general evaluation and aphasia-specific communicative accessibility evaluation tools, it was selected to be trialed by people with poststroke aphasia in the next stage of the study.

### Results of Accessibility Test

#### Participant Demographics

A total of 3 participants with a diagnosis of poststroke aphasia (2 females, 1 male) were recruited. Participants’ demographics, including what and how often they used a computer for pre- and poststroke, are included in Table 3.

### Observation Form

The level of independence each participant demonstrated while completing the first module of Moodgym, as observed by the research assistant using the observation form, is available in Table 4.

### Satisfaction Survey

Participants’ satisfaction ratings of Moodgym, as well as their comments about the program, are provided in Table 5.

**Table 1:** Summary of highest level of published evidence for each program.

Program and level of evidence		Control group	Sample size	Findings	Supports use in general population	Included people with stroke
**Moodgym (e-hub health)**						
	I [44] (meta-analysis)	N/A^a^	11 studies	Small effect size for improving symptoms of depression. Nonsignificant effect size when adjusted for potential publication bias.	✓^b^	N/A
	II [56] (RCT^c^—included in above meta-analysis)	AC^d^(online mental health information package)	340 and 219 participants completed the PHQ-9^d^ at 6 and 12 weeks follow-up.	No significant difference between Moodgym and AC in terms of psychological outcomes or service use, although improvement to subthreshold levels of depression seen in nearly half the participants in both groups at 6-week follow-up.	X^e^	✓
**E-couch (e-hub health)**						
	II [46] (RCT)	AC (online health information package)	487 participants completed the postintervention assessment.	Small but robust improvement in depression symptoms in treatment group relative to AC post intervention.	✓	✓
	II [47] (RCT)	Control website with delayed access to e-couch	209 and 176 participants completed the 6 and 12-month follow-up assessment.	E-couch was effective relative to control at post intervention but not at 6-month follow-up.	✓	X
	II [48] (RCT)	Moodgym	549 and 336 participants completed the postintervention and follow-up assessments.	Significant reduction in depression symptoms at post intervention and 6-month follow-up for both e-couch’s CBT^f^ and IPT^g^ modules and both were noninferior to Moodgym.	✓	X
**myCompass (Black Dog Institute)**						
	II [45] (RCT)	AC and WLC^h^	449 and 350 participants completed the postintervention and 3-month follow-up.	Reduction in depression symptoms relative to both control conditions post intervention. Participants in AC group showed gradual reductions in depression symptoms during postintervention stage and scores did not differ from the myCompass group at follow-up.	✓	X
**The Wellbeing Course (MindSpot)**						
	II [49] (RCT)	Mood Course^i^	229 participants completed the 24-month follow-up assessment.	Consistent reductions in MDD^j^ symptoms across conditions post intervention and 24-month follow-up.	✓	X
	II [50] (RCT)	Wellbeing course with and without automated emails compared with WLC^k^	219 and 199 participants completed the postintervention and 3-month follow-up assessments.	Reductions in symptoms of anxiety and depression relative to WLC at post intervention and 3-month follow-up.	✓	X
	II [51] (RCT)	WLC	77 participants in total, 38 of which had depression.	Reduced depression symptoms post intervention and maintained at 3-month follow-up.	✓	X
	II [52] (RCT)	Social Confidence Course^i^	172 and 170 participants completed the postintervention and 24-month follow-up assessment. 87 of these participants had depression symptoms	Consistent reduction in comorbid depression^l^ symptoms across conditions postintervention and at 24-month follow-up.	✓	X
	II [54] (RCT)	The Panic Course^i^	122 and 111 participants completed the postintervention and 24-month follow-up assessment. 38 of these participants met the diagnostic criteria for MDD.	Consistent reduction in comorbid depression^l^ symptoms across conditions over 24-month follow-up.	✓	X
	II [53] (RCT)	The Worry Course^i^	282 and 260 pts completed the postintervention and 24-month follow-up assessment. 157 participants had depression symptoms	Consistent reduction in comorbid depression^l^ symptoms across conditions post intervention and at 3-month follow-up. Treatment group’s depression symptoms slightly improved relative to AC from 3- to 12-month follow-up.	✓	X
OnTrack—Alcohol and Depression (Queensland University of Technology)^m^		N/A	N/A	N/A	N/A	N/A
OnTrack—Depression (Queensland University of Technology)^m^		N/A	N/A	N/A	N/A	N/A
Depression Center 4.0 (Evolution Health)^m^		N/A	N/A	N/A	N/A	N/A
Getselfhelp (Carol Vivyan)^m^		N/A	N/A	N/A	N/A	N/A

^a^N/A: not applicable.

^b^✓: yes.

^c^RCT: randomized controlled trial.

^d^AC: attention-control.

^e^Patient Health Questionnaire-9.

^f^X: no.

^g^CBT: cognitive behavioral therapy.

^h^IPT: interpersonal therapy.

^i^Developed specifically for the study.

^j^MDD: major depressive disorder.

^k^WLC: waitlist-control.

^l^Depression as a secondary outcome.

^m^No published evidence.

**Table 2:** Program evaluation scores and percentages.

Program	General evaluation score (N=19), n (%)^a^	Aphasia-specific evaluation score (N=20), n (%)^a^
OnTrack—Alcohol and Depression	16 (84)	17 (85)
Moodgym	16 (84)	16 (80)
myCompass	16 (84)	13 (65)
OnTrack—Depression	15 (79)	16 (80)
Wellbeing Course (Demo version)	14 (74)	15 (75)
e-couch	13 (68)	15 (75)
Depression Center 4.0	13 (68)	13 (65)
Getselfhelp	5 (26)	11 (55)

^a^Percentages were rounded up/down to the nearest whole number.

**Table 3:** Participant demographic data.

Demographic characteristics		Participant 1	Participant 2	Participant 3
Age in years		77	57	46
Time since stroke		3 years, 2 months	6 years, 4 months	6 years, 4 months
Severity of aphasia		Mild	Moderate	Severe
Highest level of education		University	University and other	University
**Previous computer use/current computer use for:**				
	Work	Weekly/N/A	Daily/N/A	Daily/N/A
	Writing letters	Fortnightly/Fortnightly	Weekly/Never	Daily/Never
	Household budgeting/financing	Weekly/Weekly	Weekly/Never	Daily/Never
	Photograph management	Never/Weekly	Monthly/Rarely	Daily/Monthly
	Home movie creation	Never/ Never	Never/Never	Monthly/Never
	PowerPoint creation	Never/Never	Weekly/Never	Weekly/Never
	Banking	Weekly/Weekly	Daily/Never	Daily/Never
	Email	Daily/Daily	Daily/Monthly	Never/Never
	Social media	Never/Weekly	Weekly/Fortnightly	Daily/Daily
	Skype	Never/Never	Monthly/Rarely	Never/Never
	General interest/web searching	Never/Weekly	Daily/Daily	Daily/Weekly
	Shopping	Never/Weekly	Monthly/Never	Daily/Never
	Entertainment	Monthly/Fortnightly	Weekly/Weekly	Daily/Daily
	Therapy—speech, language	Never/Daily	Never/Weekly	Never/Never
	Therapy—for MH difficulties	Never/Never	Never/Never	Never/Never
	Other	Nil/Nil	Nil/Nil	Nil/Nil
Type of computer/s currently used		Desktop, tablet, smartphone	Tablet	Tablet, smartphone
Needs help using a computer for:		Setting up, getting into programs, using the computer, turning computer off	Setting up	Setting up, getting into programs, using the computer, turning computer off

^a^N/A: not applicable.

**Table 4:** Participants’ levels of independence as assessed via the observation tool.

Task and rating		Participant 1	Participant 2	Participant 3
**Log into premade account**				
	Enter log-in details	Minimally independent	Minimally independent	Not at all independent
	Click on the log-in tab in upper right hand corner	Totally independent	Totally independent	Not at all independent
**Navigate program**				
	Access the “Feeling Module”	Mostly independent	Mostly independent	Not at all independent
	Use scroll bar/arrows to view all text on the page	Mostly independent	Totally independent	Not at all independent
	Use side arrows to click onto next page	Totally independent	Mostly independent	Not at all independent
**Read and understand text**				
	Read informative text	Mostly independent	Not at all independent	Not at all independent
	Read and correctly follow instructions	Minimally independent	Not at all independent	Not at all independent
**Complete interactive activities**				
	Click on the image /tab/link to access indicated information	Minimally independent	Minimally independent	Not at all independent
**Complete exercises**				
	Select yes/no answers during tasks/quizzes	Mostly independent	Minimally independent	Not at all independent
	Click on 'submit' to submit answers	Totally independent	Minimally Independent	Not at all independent
	Answer open-ended questions via text-entry field	Totally independent	Not at all independent	Not at all independent
**Finish session**				
	Log out of Moodgym	Minimally independent	Minimally independent	Not at all independent
	Exit out of Moodgym	Minimally independent	Totally independent	Minimally independent
Research assistant’s comments		Independently read informative text, but reading was slow and effortful.	All text read aloud by research assistant; difficulties completing yes/ no quizzes	All text read aloud by research assistant; mouse controlled by research assistant

**Table 5:** Results of satisfaction survey.

Question/statement in satisfaction survey	Participant 1	Participant 2	Participant 3
1. Was it easy to login?	No—I don’t think so	Yes—I think so	Yes—I think so
2. Did Moodgym look appealing?	No—I don’t think so	Neutral	Neutral
3. Was the information worded in a way that was easy to understand?	No—I don’t think so	Neutral	No—I don’t think so
4. Were the instructions worded in a way that was easy to understand?	No—I don’t think so	Yes—I think so	Yes—I think so
5. Were the words and pictures clear on the screen?	No—I don’t think so	Yes—I think so	No—I don’t think so
6. Was the text style easy to read?	Neutral	Yes—I think so	Yes—I think so
7. Was the text size easy to read?	No—I don’t think so	Yes—I think so	Neutral
8. Was there enough white space on each page?	Neutral	Neutral	Yes—I think so
9. Was it easy to find important information?	Neutral	Yes—I think so	Neutral
10. Did the pictures help you to understand the information?	No—I don’t think so	Yes—I think so	Yes—I think so
11. Was Moodgym simple to use?	No—definitely not	Neutral	No—I don’t think so
12. Could you use Moodgym without help?	No—definitely not	No—definitely not	No—definitely not
13. Moodgym looked like it was developed for someone with aphasia to use	No—definitely not	No—I don’t think so	No—I don’t think so
14. Did you enjoy using Moodgym?	No—I don’t think so	Yes—I think so	No—definitely not
15. Overall, were you satisfied with Moodgym?	No—I don’t think so	Yes—I think so	No—definitely not
16. Overall, was Moodgym easy to use?	No—I don’t think so	Neutral	No—definitely not
Comments made	“Very complex language”	“Once it was read out and explained, it was easy”	“Hard to understand”

## Discussion

### Principal Findings

This scoping review identified 8 e-mental health programs for depression that are freely available to Australian residents. Of these, 4 programs (Moodgym, e-couch, myCompass, and the Wellbeing Course) have been shown to reduce symptoms of depression within the general population. No empirical evidence was identified that specifically supported any programs’ use for stroke survivors with or without aphasia. However, it was found that e-couch and Moodgym had been evaluated using RCT methodology that included a small number of participants with stroke. Findings from a general and an aphasia-specific evaluation indicated that Moodgym was the only evidence-based program to score at least 80% (16/19 and 16/20) on both evaluations, suggesting that it may be suitable for people with poststroke aphasia. However, when trialing Moodgym, 2 of the 3 participants with aphasia required assistance for more than half of the skills assessed on the observation form, and the other participant who demonstrated higher levels of independence still gave Moodgym low satisfaction and usability scores on the posttrial satisfaction survey. Therefore, despite fulfilling majority of the general evaluation and aphasia-specific evaluation criteria, Moodgym was still found to be unsuitable for people with poststroke aphasia. This also suggests that the aphasia-specific criteria used to initially assess communicative accessibility may not have been sensitive enough to detect suitability for people with poststroke aphasia and thus should be revised if it is to be used in any future research.

Consistent with previous findings in the literature, this study found that the programs’ content was usually generic for all users and that the programs themselves tended to be based on CBT, either alone or in combination with other approaches [28,29]. This may be explained by the fact that CBT is the most researched psychotherapy and one of the first forms of psychotherapy to be established as evidence-based [57]. Furthermore, because of its structured protocols and ability to be manualized, CBT lends itself well to self-help e-mental health platforms [58]. This study also builds on the findings of the previous scoping review on e-mental health programs for depression [28]. First, only a few programs were found to send out completion reminders via email and/or text message. This strategy has been found to increase adherence to medical treatment [59] and may be especially useful for e-mental health programs that tend to have high dropout rates [60]. Second, no program was found to have a companion app able to be purchased at an official Australian Apple or Android app store at the time of this review. Previous research investigating mobile computing technology and aphasia [33] suggests that an advantage of mobile computer apps is their ability to be adapted for people with disabilities. For example, buttons on touch screens can be customized, unlike the physical mouse and keyboard of desktop computers, and apps and touch interfaces can be changed to suit users’ vision and mobility needs, as there is no set button size [33]. This may be particularly relevant to stroke survivors, who often face upper extremity motor impairment post stroke [61]. Furthermore, mobile computer devices such as the Apple iPad may offer more accessibility options such as predictive text, switch control, and VoiceOver.

While this review did not identify any new e-mental health programs for depression released since the previous scoping review [28], it did identify new empirical evidence for some of the programs. Namely, this study identified a meta-analysis supporting the efficacy of Moodgym [44] and RCTs supporting the use of myCompass [45] and the Wellbeing Course [49,52-54] by the general population. Unfortunately, this study found that there is currently a lack of high-quality research investigating the efficacy of e-mental health programs for stroke survivors with and without aphasia. Furthermore, the studies that did include stroke participants failed to specify the presence or absence of people with poststroke aphasia within the included stroke samples. This supports recent findings that people with poststroke aphasia are often excluded or inadequately reported on as a subsample within stroke populations in mental health research [62]. As suggested by Baker and colleagues [62], it is important that mental health studies include a minimum dataset for people with poststroke aphasia included in a study (ie, report number of people with poststroke aphasia and severity and nature of communication difficulties) and conduct subgroup analyses. Failure to do so will hinder the progression of research for people with poststroke aphasia in this area. This is of particular importance as the prevalence of psychological conditions among people with stroke (with and without aphasia) is likely to continue to increase alongside an aging population [63,64] and accompanied increased incidence of stroke [65].

People with poststroke aphasia have previously been identified as being victims of *digital exclusion* as a result of their language deficits, age- and stroke-related changes, and lack of premorbid computer and internet skills often attributed to their generally older age [33,66]. However, consistent with previous findings [39], the results of the computer use survey indicated a high level of computer usage by the participants with aphasia before and after their stroke. This aligns with other research, which suggests that tomorrow’s elders with disabilities will generally have more access to and increased proficiency with wireless technologies than their predecessors [67]. Thus, it can be assumed that as technology continues to advance to meet the growing needs of consumers, and as the number of older adults with digital literacy increases, it is likely that less people with poststroke aphasia will face digital exclusion than in previous years [67].

The communicative accessibility of the reviewed programs for people with poststroke aphasia was initially evaluated against an aphasia-specific evaluation tool. Aphasia-specific evaluation scores ranged from 55% (11/20) to 85% (17/20), with only 3 programs scoring 80% or more (Moodgym, OnTrack—AD, and OnTrack—Depression). Hence, these findings suggest that while all programs incorporated design features consistent with published aphasia-friendly guidelines and other recommendations, the communicative accessibility of e-mental health programs could potentially be improved to render these programs more appropriate to people with poststroke aphasia. Findings indicated that many programs might be improved by greater use of visuographic supports. For instance, pictures, which directly support the text, have been found to enhance reading comprehension in people with aphasia [68,69]. For example, it was noted that Moodgym’s “yes” or “no” checkbox quizzes, which did not contain pictorial support, were particularly difficult for 1 participant with poststroke aphasia to complete. Unreliable “yes” or “no” responses is a common symptom of aphasia [70]. Therefore, such exercises could be made more aphasia-friendly by including pictorial supports such as a picture of a tick next to the “yes” option and a picture of a cross next to the “no” option for each item. It should also be noted that poststroke aphasia can differentially affect one’s language modalities (speaking, understanding, reading, and writing, etc) [71], resulting in people with poststroke aphasia having different language “profiles,” which may make it easier or harder for them to access e-mental health support. Therefore, incorporating a wider variety of media types, including videos, animation, graphics, and audio, would likely increase the accessibility of the programs to meet the differing communicative needs of people with poststroke aphasia.

As assessed via the aphasia-specific communicative accessibility evaluation tool, this study found that none of the evaluated programs had an average readability level of 5 or lower, which is the level recommended for people with aphasia [38]. As reported by the participants with aphasia, the language used in Moodgym, which had a readability of 7.9, was complex and hard to understand, and 2 participants required all text to be read aloud to them. Furthermore, prior literature has found that reliable Web-based health information is often presented at reading levels too high for the general population as it is [72-76]. Therefore, reducing the text readability level of e-mental health programs should increase their accessibility not only to people with poststroke aphasia but also to the general population as a whole.

While appropriate readability levels of written materials are important, there are other formatting characteristics that can facilitate reading comprehension in people with poststroke aphasia. These include use of large font (ie, 14-point or larger) [77], generous spacing between lines to maximize white space [77], the use of headings and bolding to make important information stand out [34], and the use of bullet points and numbers to clearly establish key points [34]. All reviewed programs were found to use headings, bolding, bullet points and numbering, although to differing degrees. However, some programs could enhance their accessibility by using more white space between lines of text (ie, 1.5 spacing) to make content appear more appealing and readable [77] and by using bolding to highlight key information [34].

### Limitations and Direction for Further Research

Despite the authors’ efforts to be systematic and inclusive in their search for e-mental health programs for depression, it is possible that some programs were missed. The dynamic nature of Web content also means that the identified programs may eventually be changed or discontinued, and new programs will likely be developed in the future. Furthermore, this study excluded paid programs (n=9) and programs for which the authors were not granted research access (n=4). Inclusion of these programs may have yielded different results and thus resulted in different recommendations.

The aphasia-specific search terms used in the search strategy were informed by suggestions from clinicians and academics that work with people with poststroke aphasia, rather than people with poststroke aphasia themselves. Future studies investigating how people with poststroke aphasia use the internet to find e-mental health programs, and if their search terms are successful in locating the same programs identified in this review, may also be of benefit.

This small trial of people with poststroke aphasia was helpful in determining whether the highest rated e-mental health program was communicatively accessible to people with poststroke aphasia, or not. However, a larger trial of people with poststroke aphasia is paramount in determining which aspects of current e-mental health programs are most accessible, and which aspects need improvement. The use of think-aloud studies, whereby participants’ experiences of the program are captured using multimodal communication, may be one way to identify accessibility facilitators and barriers of such e-mental health programs. Furthermore, if e-mental health programs are to be redesigned or future programs developed specifically for people with poststroke aphasia, end users (ie, people with aphasia) should participate in the design and development process to ensure usability within the target population. It is acknowledged that participatory design approaches rely heavily on effective communication between the participants and designers and thus may be challenging for people with poststroke aphasia [78]. However, there are numerous examples of people with poststroke aphasia successfully partaking in participatory design studies to enhance the usability of aphasia-specific programs [19,78-82].

### Conclusions

E-mental health services offer convenient and cost-effective interventions that have an ability to reach a more diverse population than traditional face-to-face psychological interventions [21]. Thus, the next decade will likely see mental health services progress toward a digital medium, which will present numerous opportunities for both clinical practice and e-mental health research [83]. It is important that people with poststroke aphasia, a population with an increased risk of developing depression, are considered in future e-mental health research. Failure to do so may mean that people with poststroke aphasia and mild to moderate depression may not be offered e-mental health treatment, or the options available to them may remain inaccessible, and therefore potentially nonbeneficial to them.

## Appendix

Multimedia Appendix 1 Data extraction form.

Multimedia Appendix 2 Completed general evaluation form.

Multimedia Appendix 3 Completed aphasia-specific evaluation form.

Multimedia Appendix 4 Website characteristics of included programs.

Multimedia Appendix 5 Program characteristics of included programs.

Multimedia Appendix 6 Intervention characteristics of included programs.

## Abbreviations

CBT: cognitive behavioral therapy
cCBT: computerized cognitive behavioral therapy
CINAHL: Cumulative Index to Nursing and Allied Health Literature
IPT: interpersonal therapy
RCT: randomized controlled trial
OnTrack—AD: OnTrack—Alcohol and Depression
