# Supplementary material for: Accessibility and Applicability of Currently Available e-Mental Health Programs for Depression for People With Poststroke Aphasia: Scoping Review
Source: J Med Internet Res. 2018 Dec 4;20(12):e291. doi: 10.2196/jmir.9864 (PMC6299232; doi:10.2196/jmir.9864)
Supplement: Multimedia Appendix 1 [file jmir_v20i12e291_app1.pdf]

Multimedia Appendix 1. Data extraction form.

Main Category	Subcategory	Item
**Website characteristics**	**Origin**	
		Country of origin
	***Organisational affiliation**	
		[a]Is it affiliated with an organisation (Yes, no- if yes who?)
	**Accessibility**	
		Registration (yes/no—if yes, how?)
		Log-in available on website (yes/no)
		[a]International accessibility
		[b]Mobile app available (yes/no)
		[a]Was there paid access to certain content?
	**Credibility**	
		Advertisements (yes/no- if yes, relevant vs irrelevant)
		Presented contact details (yes/no)
		Specified authorship (yes/no)
		Terms of use specified (yes/no)
		Privacy notice specified (yes/no)
**Program characteristics**	**Intervention focus**	
		Target [c]depression issue
		Target audience
	**Intervention design**	
		Therapist support (yes/no—if yes, specify)
		Suggested or set treatment length
		Number of modules
	**Intervention delivery**	
		Presentation format
**Intervention characteristics**	**Therapeutic approach**	
		CBT; others (specify)
		Other therapeutic elements
**Main Category**	**Subcategory**	**Item**
	**Intervention features**	
		Worksheets (yes/no—if yes, specify format)
		Mood or symptom monitoring (yes/no)
		Diary (yes/no)
		Forum (yes/no)
		Other features (yes/no—if yes, specify)
**Empirical evidence**	**Empirical evidence for program efficacy**	
		Scrutinized program website for relevant information, [a]searched the

		following data bases: PubMed, CINAHL, Cochrane Library, and Web of Science, contacted the author
	[a]**Empirical evidence for program efficacy after stroke**	
		[a]Scrutinized program website for relevant information, searched the following data bases: PubMed, CINAHL, Cochrane Library, and Web of Science, contacted the author
	[a]**Empirical evidence for program efficacy for people with post-stroke aphasia**	
		[a]Scrutinized program website for relevant information, searched the following data bases: PubMed, CINAHL, Cochrane Library, and Web of Science, contacted the author

[a]Subcategory, item or item detail was added to original data extraction tool [32] to form the adapted data extraction tool for the current study.
[b]Changed from: ‘Mobile phone rendering (yes/no)’ [32].
[c]Changed from ‘anxiety’ [32].
Reproduced with permission.
