# Supplementary material for: Accessibility and Applicability of Currently Available e-Mental Health Programs for Depression for People With Poststroke Aphasia: Scoping Review
Source: J Med Internet Res. 2018 Dec 4;20(12):e291. doi: 10.2196/jmir.9864 (PMC6299232; doi:10.2196/jmir.9864)
Supplement: Multimedia Appendix 2 [file jmir_v20i12e291_app2.pdf]

Multimedia Appendix 2. Completed general evaluation form.

General evaluation scores									
**Main category**	**Question**	**MG**	**e-couch**	**myCom**	**OT – D**	**OT - A&D**	**WB Course**	**GSH**	**Dep Center**
**Website characteristics**									
	Was country of origin stated?	Y	Y	Y	Y	Y	Y	N	N
	Was a unique user name or password provided to users?	Y	Y	Y	Y	Y	Y	N	Y
	Were the names and credentials of authors present?	Y	Y	N	Y	Y	Y	N - N cred.	N
	Were contact details provided?	Y	Y	Y	Y	Y	Y	Y	Y
	Were the Terms of Use specified?	Y	Y	Y	Y	Y	Y	N	Y
	Was a Privacy Notice specified?	Y	Y	Y	Y	Y	Y	Y	Y
	Was evidence for the program provided to the user (i.e., attrition data/success rate/completion rate/# of users in the program/testimonials)?	Y	N	N	N	Y	Y	N	Y
	Was a mobile app version of the program available?	N	N	N	N	N	N	N	N
**Program characteristics**									
	Were the primary focus/goals/objectives of the intervention stated?	Y	Y	Y	Y	Y	Y	N	Y
	Was the patient group or target mental health issue specified?	Y	Y	Y	Y	Y	Y	Y	Y
	Was the number of modules or time to complete each module stated?	Y	N	Y	Y	Y	Y	N	Y
	Was the intervention tailored to the user or was it generic for all users?	N	N	Y	N	N	Y	N	N
	Did the program offer multimedia content delivery (i.e., a combination of text, video, graphics, and audio formats)?	Y	Y	Y	Y	Y	-	N	Y
	Was the program easy to navigate?	Y	N	Y	Y	Y	-	N	Y
	Did the program send	N	N	Y	Y	Y	N	N	N

	completion reminders?								
	Were text-entry fields present?	Y	Y	Y	Y	Y	-	N	Y
**Intervention characteristics**									
	Was the model of change (i.e., type of therapy utilized) defined/stated?	Y	Y	Y	Y	Y	Y	Y	Y
	Was information on what is covered in the intervention modules provided (i.e., names of modules or a short description)?	Y	Y	Y	Y	Y	Y	Y	Y
**Empirical evidence**									
	Has the program been empirically validated?	Y	Y	Y	N	N	Y	N	N
**Total score (out of 19):**									
		**16**	**13**	**16**	**15**	**16**	**14**	**5**	**13**

Abbreviations: Dep Center, Depression Center 4.0; GSH, getselfhelp CBT Self Help Course; MG, moodgym; myCom, myCompass; N, no; OT – A&D, OnTrack – Alcohol and Depression course; OT – D, OnTrack – Depression course; WB course, the MindSpot Clinic’s Wellbeing Course (demo version); Y, yes.
Symbols: -, could not be evaluated.
General evaluation form reproduced with permission.
