# Supplementary material for: Accessibility and Applicability of Currently Available e-Mental Health Programs for Depression for People With Poststroke Aphasia: Scoping Review
Source: J Med Internet Res. 2018 Dec 4;20(12):e291. doi: 10.2196/jmir.9864 (PMC6299232; doi:10.2196/jmir.9864)
Supplement: Multimedia Appendix 3 [file jmir_v20i12e291_app3.pdf]

Multimedia Appendix 3. Completed aphasia-specific evaluation form

Main category	Question	MG	e-couch	myCom	OT-D	OT - A&D	WB Course	GSH	Dep Center
**Vocab & syntax**									
	Was the reading level 5 or lower?[a]	N (7.9)	N (6.5)	N (5.7)	N (5.9)	N (5.4)	N (8.2)	N (9.0)	N (8.4)
**Screen clarity**									
	Does the colour of the font contrast the colour of the BG?	Y	Y	Y	Y	Y	Y	Y	Y
**Formatting**									
	Was a large font size used?[b]	Y	N	Y	N	N	Y	Y	N
	Was there white space of at least 4mm between lines of text?[c]	N	N	N	Y	Y	Y	N	N
	Were bullet points/ numbering used to establish key points?[d]	Y	Y	Y	Y	Y	Y	Y	Y
	Were headings used to make important info standout?	Y	Y	Y	Y	Y	Y	Y	Y
	Was bolding used to highlight important info?	Y	Y	Y	Y	Y	Y	Y	Y
**Graphics**									
	Were graphics used?	Y	Y	Y	Y	Y	Y	Y	Y
	Did the graphics add meaning to or enhance the content of the text?	Y	Y	Y	Y	Y	Y	Y	Y
	Were graphics labelled?	Y	Y	N	Y	Y	N	N	Y
**Navigation**									
	Did the website have simple	Y	Y	Y	Y	Y	Y	N	Y

	menu hierarchies?								
	Were modules/ lessons presented in clearly defined steps?[e]	N	N	N	N	N	Y	N	Y
**Interface design**									
	Was there a stable interface?[f]	Y	Y	Y	Y	Y	Y	N	Y
	Was there a visually simplistic interface design?	Y	Y	Y	Y	Y	Y	N	N
**Media type**									
	Did the MT include audio?	Y	Y	N	Y	Y	-	N	N
	Did the MT include text?	Y	Y	Y	Y	Y	Y	Y	Y
	Did the MT include pictures/ graphics?	Y	Y	Y	Y	Y	Y	Y	Y
	Did the MT include video?	N	N	N	Y	Y	-	Y	N
	Was animation present?	Y	Y	N	N	Y	-	N	N
	Was the MT a combination of more 2 or more of the above categories?	Y	Y	Y	Y	Y	Y	Y	Y
	**Score (from 20):**	**16**	**15**	**13**	**16**	**17**	**15**	**11**	**13**

Abbreviations: BG, background; Dep Center, Depression Center 4.0; GSH, getselfhelp CBT Self Help Course; info, information; MG, moodgym; myCom, MT, media type; myCompass; N, no; OT - A&D, OnTrack – Alcohol and Depression course; OT - D, OnTrack – Depression course; vocab, vocabulary; WB course, the MindSpot Clinic's Wellbeing Course (demo version); Y, yes.

Symbols: -, could not be evaluated.

[a]using Flesch-Kincaid grade levels

[b]14-point or larger

[c] As measured with ruler on a 15.6 inch diameter laptop screen; 100% webpage zoom; screen resolution set at 1366 x 768

[d] rather than embedding points in paragraphs of running pose

[e]i.e., numbered

[f]i.e., contents of screen were predictable, did not change order or contain pop-ups
