# Supplementary material for: Accessibility and Applicability of Currently Available e-Mental Health Programs for Depression for People With Poststroke Aphasia: Scoping Review
Source: J Med Internet Res. 2018 Dec 4;20(12):e291. doi: 10.2196/jmir.9864 (PMC6299232; doi:10.2196/jmir.9864)
Supplement: Multimedia Appendix 4 [file jmir_v20i12e291_app4.pdf]

Multimedia Appendix 4. Website characteristics of included programs.

	**e-couch**	**GSH**	**WB Course**	**MG**	**myCompass**	**OnTrack – AD**	**OnTrack - Dep**	**Dep Centre**
Country of origin	Aus	UK	Aus	Aus	Aus	Aus	Aus	Unknown
Organisation affiliation	Beyond-Blue, ANU, Aus Gov Dep of Health & Aging	N	Macq, beyondblue Mindhealth-connect, funded by the Aus Gov	eHub Health, ANU	Blackdog Institute, funded by the Commonwealth Dep of Health	QUT, QLD Gov	QUT, QLD Gov	Evolution Health
Registration	Free online	N	Free online	Free online	Free online	Free online	Free online	Free online
Log-in available?	Y	N	Y	Y	Y	Y	Y	Y
International accessibility	Y – free of charge world-wide	Y – free of charge world-wide	N – can only be accessed by Aus residents	N - Non-Aus users to pay subscription fee; organisational/ institutional subscriptions also available	N – can only be accessed by Aus residents	Y – free of charge world-wide	Y – free of charge world-wide	Y – free of charge world-wide
Mobile app available?	N	N	N	N	N	N	N	N
Paid access to certain content?	N	Y – to mp3 downloads	N	N	N	N	N	N
Ads	N	Y - relevant (i.e., for self-help books) & irrelevant (i.e., for Amazon)	N	N	N	N	N	N
Contact details presented?	Y	Y	Y	Y	Y	Y	Y	Y
Specified authorship?	Y	Y	Y	Y	Y	Y	Y	N
Terms of use specified?	Y	N	Y	Y	Y	Y	Y	Y
Privacy notice	Y	Y	Y	Y	Y	Y	Y	Y

specified?								
Abbreviations: ANU, Australian National University; Aus, Australia; dep, department; Dep Center, Depression Center 4.0; gov, government; GSH, getselfhelp CBT Self Help Course; Macq, Macquarie University; MG, Moodgym; N, no; OnTrack – AD, OnTrack – Alcohol and Depression; OnTrack – Dep, OnTrack – Depression; QLD, Queensland; QUT, Queensland University of Technology; WB Course, Wellbeing Course; Y, yes.								
