# Supplementary material for: Accessibility and Applicability of Currently Available e-Mental Health Programs for Depression for People With Poststroke Aphasia: Scoping Review
Source: J Med Internet Res. 2018 Dec 4;20(12):e291. doi: 10.2196/jmir.9864 (PMC6299232; doi:10.2196/jmir.9864)
Supplement: Multimedia Appendix 5 [file jmir_v20i12e291_app5.pdf]

Multimedia Appendix 5. Program characteristics of included programs.

	Target issue	Guided	Tx length[a]	No. of mods	Media type
**Dep Center**	- Dep	Yes	12-16 wks	18 sessions	- Text,<br>- Graphics<br>- TEF
**e-couch**	- Dep	No	Not specified	- CBT = 3 mods<br>- IPT = 4 mods<br>- PS = 5 steps<br>- Physical activity mod = no specific mods<br>- Relax mod = 7 steps	- Audio<br>- Text<br>- Graphics<br>- AMN<br>- TEF
**GSH**	- Anxiety<br>- Dep<br>- Anger	No	Not specified	- 7 steps	- Text<br>- Graphics<br>- Video (YouTube videos)
**MG**	- Dep<br>- Anxiety	No	Not specified	- 5 mods	- Audio<br>- Text<br>- Graphics<br>- AMN<br>- TEF
**myCompass**	- Dep<br>- Anxiety<br>- Stress	No	Different for each mod. Most approx. 2-3 wks	14 mods - 12 which are related to dep	- Text<br>- Graphics<br>- TEF
**OnTrack – AD**	- Low mood & alcohol problems	No	- 8 wks	- 7 mods (inc intro mod)	- Audio<br>- Text<br>-Graphics<br>- Video<br>-AMN<br>- TEF
**OnTrack - D**	- Dep	No	- 8 wks	- 6 mods (inc intro mod)	- Audio<br>- Text<br>-Graphics<br>- Video<br>- TEF
**WB Course**	- Anxiety<br>- Worry<br>- Stress<br>- Dep<br>- Low mood	Yes	- 8 wks	- 5 mods	- Text<br>- Graphics[b]

Abbreviations: AMN, animation; approx.., approximately; CBT, cognitive behavioural therapy; dep, depression; Dep Center, Depression Center 4.0; GSH, getselfhelp CBT Self Help Course; inc, including; intro, introduction; IPT; interpersonal therapy; MG, Moodgym; mod/s, module/s; N, no; OnTrack – AD, OnTrack – Alcohol and Depression; OnTrack – Dep, OnTrack – Depression; PS, positive psychology; TEF, text-entry fields; Tx, treatment; WB Course, Wellbeing Course; wks, weeks;

[a] As suggested or recommended by the program.

[b] Media type could only be determined for the Demo version of the program.
