# Supplementary material for: Accessibility and Applicability of Currently Available e-Mental Health Programs for Depression for People With Poststroke Aphasia: Scoping Review
Source: J Med Internet Res. 2018 Dec 4;20(12):e291. doi: 10.2196/jmir.9864 (PMC6299232; doi:10.2196/jmir.9864)
Supplement: Multimedia Appendix 6 [file jmir_v20i12e291_app6.pdf]

Multimedia Appendix 6. Intervention characteristics of included programs.

	**Therapeutic approach**	**Other therapeutic elements**	**Intervention features**
**e-couch**	Can choose from different "toolkits": CBT, IPT, PS, physical activity, relaxation	- E-mental health literacy<br>- Relaxation<br>- Physical exercises<br>- Goal setting	- Worksheets<br>- Mood & symptom monitoring<br>- Diary<br>- Relaxation audio
**GSH**	- CBT	- E-mental health literacy	- Worksheets<br>- Relevant YouTube videos<br>- Purchasable mp3 audio files (optional). Can be used to reinforce therapy, or as a stand-alone self-help therapy technique
**Dep Center**	- CBT	- E-mental health literacy	- Worksheets<br>- Mood & symptom monitoring<br>- Medication & dep glossary<br>- Skills quiz after each mod<br>- A *Helping Fellow Members* link which provides info re: how to support fellow members
**MG**	- CBT<br>- IPT	- Relaxation exercises<br>- Goal setting<br>- Experience stories (of fictional characters)	- Worksheets<br>- Mood & symptom monitoring<br>- Down-loadable relaxation audio
**myCompass**	- CBT<br>- IPT<br>- PS	- Relaxation<br>- Experience stories (of real people)<br>- E-mental health literacy & education on other life topics<br>- Goal setting - setting SMART goals module	- Worksheets<br>- Mood & symptom monitoring<br>- Diary<br>- Motivational text messages<br>- Recommended for further reading (i.e., books)
**OnTrack – AD**	- CBT<br>- Motivational therapy<br>- Meditation mindfulness	- E-mental health literacy<br>- Goal setting	- Mood & symptom monitoring<br>- Diary<br>- Asks for program feedback<br>- "Fun and success card" whereby the user records the enjoyable and/or successful things done each day
**OnTrack - D**	- CBT<br>- Motivational therapy<br>- Meditation mindfulness	- E-mental health literacy<br>- Goal setting	- Mood & symptom monitoring<br>- Diary<br>- Asks for program feedback
**WB Course**	- CBT	- E-mental health literacy	- Worksheets<br>- Mood & symptom monitoring<br>- Helpful resources i.e., *Good Sleep Guide* PDF<br>- Contact w a MindSpot therapist

Abbreviations: CBT, cognitive behavioural therapy; Dep Center, Depression Center 4.0; GSH, getselfhelp CBT Self Help Course; IPT; interpersonal therapy; MG, Moodgym; OnTrack – AD, OnTrack – Alcohol and Depression; OnTrack – Dep, OnTrack – Depression; PS, positive psychology; w, with; WB Course, Wellbeing Course.
